# Systemic Lupus Erythematosus Mimicking a Possible Eating Disorder

**DOI:** 10.7759/cureus.83248

**Published:** 2025-04-30

**Authors:** Landon Bruich, Dustin Williford, Jacob Filipek

**Affiliations:** 1 Pediatrics/Hospital Medicine, University of Arkansas for Medical Sciences, Little Rock, USA

**Keywords:** eating disorder, generalized weakness, intermittent hematochezia, loss of weight, systemic lupus erythema

## Abstract

Systemic lupus erythematosus (SLE) is an autoimmune disease that presents with a wide range of symptoms due to its ability to affect multiple organ systems. We present a case report of a 17-year-old female with a chief complaint of a four-month history of weight loss and generalized weakness, along with a two-month history of intermittent hematochezia. Features of the patient’s initial presentation made the treatment team suspicious of anorexia nervosa. However, further workup led the team to a diagnosis of SLE. This case exemplifies a rare presentation of lupus that initially mimicked an eating disorder and did not develop classic lupus findings until months after the disease process began.

## Introduction

Rapid weight loss, low BMI, features of malnutrition, and secondary amenorrhea in adolescent women should raise concern about the possibility that the patient has an eating disorder such as anorexia nervosa (AN) [1]. To be diagnosed with AN, low BMI is only one part of the necessary criteria; the remaining features include the patient possessing a fear of gaining weight or using methods such as restricting and purging to prevent weight gain along with the patient having a disproportionate value on weight and body shape [1-3]. These criteria help differentiate AN from medical conditions that can present similarly, such as endocrine, gastrointestinal, and neoplastic diseases, or a major depressive episode [2,3].

Similar to AN, systemic lupus erythematosus (SLE) commonly affects young women and can present with nonspecific symptoms such as weight loss and fatigue [4]. Despite the common, vague symptoms in the two diseases, SLE is not typically included on lists of differentials when AN is being diagnosed [2,3]. This is likely due to SLE typically having classic features not seen in AN, such as fever, rash, and arthritis. Additionally, multiple organ systems can be impacted by SLE and lead to a variety of findings such as cytopenia, serositis, and kidney disease [5]. Our case report aims to provide an example of how SLE may not initially exhibit classic features and instead presents more consistently with AN until more typical features develop later.

This article was previously presented at the 2023 Southern Society for Pediatric Research’s Southern Regional Meeting on February 3, 2023.

## Case presentation

A 17-year-old female with a self-reported history of anemia of unknown origin presented to the ED with fatigue, generalized muscle weakness, a 6.5 kg weight loss, 11 months of amenorrhea, and two months of hematochezia. Her father stated that the symptoms began approximately four months prior, around the time of her immigration from Guam to the United States. She continued to progressively worsen to the point where she was so weak that she had difficulty getting up from the couch, prompting them to visit the ED. She takes no daily medications, and family history was noncontributory. Medical records from Guam only noted a possible past diagnosis of rheumatic heart disease. Upon initial evaluation, the patient was tachycardic to the 120s but afebrile at 99.4°F and normotensive at 125/84. Her BMI was 16.0 kg/m², placing her below the 1st percentile for her age and sex. The physical exam was unremarkable, except for a flat affect. She was admitted for further workup due to the tachycardia and concern for a possible eating disorder. Initial lab results were collected, revealing several notable findings, including those related to malnutrition (Table 1).

**Table 1:** Initial remarkable laboratory findings BUN: blood urea nitrogen, ESR: erythrocyte sedimentation rate

Lab	Value	Reference range
Hemoglobin	6.0 g/dL	12.0-16.0 g/dL
Platelets	123 K/uL	150-400 K/uL
BUN	34 mg/dL	7-18 mg/dL
Creatinine	1.35 mg/dL	0.6-1.2 mg/dL
Calcium	7.3 mg/dL	8.5-10.7 mg/dL
ESR	105 MM/HR	0.0-20.0 MM/HR
Prealbumin	<5.0 mg/dL	12.0-42.0 mg/dL
Albumin	2.6 g/dL	3.7-5.6 g/dL
Vitamin B1	2.0 nmol/L	4.0-15.0 nmol/L
Vitamin C	13.0 umol/L	23.0-114.0 umol/L

**Figure 1:**
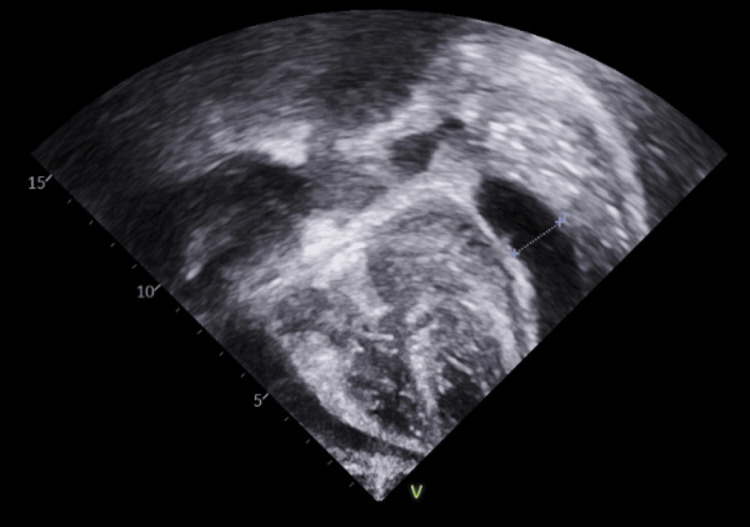
TTE showing pericardial effusion denoted by Xs and line TTE: transthoracic echocardiogram

On the first day of admission, esophagogastroduodenoscopy and colonoscopy were performed to evaluate the hematochezia, with no concerning findings. Echocardiogram to evaluate possible rheumatic heart disease showed a structurally normal heart with a mild pericardial effusion (Figure 1). An eating disorder specialist was consulted and determined that an eating disorder was unlikely despite the low BMI, as the patient stated that she felt “too skinny” and wanted to gain weight rather than intentionally lose weight. She said her appetite had decreased, but she still ate two meals and had snacks each day. She denied any purging. A rheumatologic condition, particularly SLE, was the most likely etiology of the patient’s features. The new laboratory evaluation consisted of measuring complement components, sending off ANA and dsDNA titers, and evaluating for renal involvement. C3 and C4 were low at 21 mg/dl (reference range: 70-206 mg/dl) and 3.9 mg/dl (reference range: 11.0-61.0 mg/dl). Urine protein-to-creatinine ratio was 1.48 g/day (reference range: <0.2 g/day). The patient remained hospitalized and was stable until hospital day 6, when she developed acute shortness of breath, was tachypneic, was tachycardic at 141 beats per minute, had decreased peripheral pulses, and had muffled heart sounds. An urgent bedside echocardiogram showed a 16 mm pericardial effusion and variations in mitral and tricuspid valve inflow with respiration, suggesting cardiac tamponade. Pericardiocentesis was performed, and the patient was stabilized and transferred to the pediatric intensive care unit. Soon after, the ANA titer returned >1:2560 (reference range: <1:80); the dsDNA antibody IgG ELISA result was 298 IU (reference range: 0-24 IU), which is highly specific for SLE.

With the diagnosis now clear, the patient was started on 30 mg/kg of IV methylprednisolone for three days, then transitioned to 60 mg daily of oral prednisone. Mycophenolate mofetil 750 mg twice a day and hydroxychloroquine 200 mg daily were also initiated. Laboratory results one month later showed improved hemoglobin and platelet counts at 10.7 g/dL and 225 K/μL, respectively. The protein-to-creatinine ratio improved to 33.3:60.0 mg/dL, suggesting decreased renal involvement. C3 and C4 levels returned to the reference range at 123 mg/dL and 23.9 mg/dL.

She had no new development of pericardial effusion. Her BMI increased from 16 to 24. Regarding symptoms, the patient experienced significant improvement in energy and strength and was eventually discharged from the hospital. She continues to be maintained on mycophenolate mofetil and hydroxychloroquine, with regular follow-up in rheumatology and nephrology clinics. A repeat echocardiogram showed resolution of her pericardial effusion. She has had no further admissions related to her condition and is being transitioned to adult care for continued follow-up.

## Discussion

This case of SLE in an adolescent female initially presented with fatigue, muscle weakness, weight loss, secondary amenorrhea, and signs of malnutrition, all of which could be explained by AN. No classic symptoms of SLE were initially present, including no fevers, arthralgias, or rash [1,6]. A retrospective chart review identified seven patients with coexisting SLE and AN. However, it did not explore the presence of worrisome signs related to AN in patients with SLE who did not have coexisting AN [7]. Although the patient in this case did not meet all the diagnostic criteria of AN, it provided an example where features consistent with AN preceded the development of the classic symptoms of SLE in a patient with SLE.

The presence of AN features prior to the recognition of SLE features can delay diagnosis and treatment initiation of SLE. In a case report with coexisting AN and SLE, there were two flares of SLE before it was recognized that the patient had both conditions [4]. In the retrospective review, the development of AN symptoms occurred by a median of 19.7 months before the patients were diagnosed with SLE [7]. The authors of this review go so far as to say that the development of AN may be an early manifestation of SLE [7]. In this case, which initially mimicked AN, the team continued to investigate other etiologies for the patient’s presentation, leading to a diagnosis of SLE without AN.

This suspicion of SLE helped the team recognize and intervene rapidly in the case of cardiac tamponade secondary to a pericardial effusion. Due to the diagnosis of SLE, the patient received methylprednisolone in addition to pericardiocentesis. The addition of methylprednisolone could have prevented the recurrence of tamponade and helped reduce the chance that the patient would need a repeat pericardiocentesis or surgery to manage recurrent tamponade [8]. In addition to helping prevent tamponade, early treatment of SLE has been shown to avoid other causes of mortality, including end-stage kidney disease from lupus nephritis [8,9]. Being able to initiate early treatment of lupus is dependent upon the ability to quickly diagnose, which can be difficult in cases like this where the patient had an atypical presentation of lupus for months prior to developing more typical features.

## Conclusions

Extreme weight loss, muscle weakness, and secondary amenorrhea in adolescent females typically raise suspicion for eating disorders such as AN. Consequently, SLE is rarely considered in the differential diagnosis when evaluating a patient for AN. This case report illustrates a rare presentation where SLE can lack the classic findings of joint and skin involvement but may mimic an eating disorder in its early stages with vague symptoms, possibly delaying recognition until more characteristic signs emerge. Further evaluation and the development of new symptoms led to a diagnosis of SLE. This allowed for the rapid initiation of immunosuppressive treatment, which can help prevent life-threatening manifestations of SLE, specifically cardiac tamponade in this case. With this case, we aim to emphasize the need to maintain a broad differential that includes autoimmune disorders such as SLE in adolescent females with unexplained weight loss, fatigue, and amenorrhea, especially in the absence of classic disordered eating behaviors.
